# Effects of hesperidin on formaldehyde-induced toxicity in pregnant rats

**DOI:** 10.17179/excli2017-142

**Published:** 2017-03-30

**Authors:** Sameha Merzoug, Mohamed Lamine Toumi

**Affiliations:** 1 Department of Biology, Faculty of Natural and Life Sciences, University of Chadli Bendjedid - El-Tarf, BP 73, 36000, El-Tarf, Algeria

**Keywords:** formaldehyde, hesperidin, pathophysiology, behavior, gestation, fetal development

## Abstract

This experimental study aimed to investigate the protective effect of a bioflavonoid, hesperidin (HP), on formaldehyde (FA)-related pathophysiological and behavioral outcomes in pregnant rats and developmental aspects in their offspring. Female Wistar rats were subjected to perigestational exposure to FA (2 mg/kg/day *per os*) with a concomitant treatment with HP (50 mg/kg/day *per os*). Pregnant rats were weighed throughout gestation and tested in two behavioral paradigms (elevated plus-maze and open field) at gestational days (GD) 1, 10 and 19 to evaluate the anxiety-like behavior and locomotive alterations. Another subset of rats was decapitated at GD19 to determine the hematological profile along with cortisol, 17β-estradiol, and progesterone plasma levels. Reproductive and fetal measures and observations were also performed to check for developmental deformities. Significant body weight loss, hemato-immune decline, hormonal changes, anxiety and lethargy signs, locomotor disabilities, reproductive failure and fetal weight decrease were observed in FA-exposed rats. Treatment with HP alleviated the reproductive and fetal weight defects. Its behavioral benefits were only seen at GD1 and 10. This flavanone ameliorated some hematological parameters, decreased cortisol levels and increased 17β-estradiol rates. A potential preventive impact of HP was found against FA toxicity in pregnant rats.

## Introduction

Formaldehyde (FA) is an organic compound that naturally occurs in the form of a colorless gas. Its high toxicity and tissue-necrotic effects regarding various organs have been neatly reported in experimental animals a long time ago (Fischer, 1905[11]). Due to its multiple industrial applications, FA is readily inhaled and/or ingested by human beings who can develop a wide range of pathophysiological symptoms depending on the exposure duration (Kim et al., 2011[21]). Over the last few decades, a particular attention has been paid to reproductive and developmental outcomes among pregnant women who were exposed to amounts of FA high enough to cause both maternal and fetal defects, such as spontaneous abortion, premature birth, and congenital malformations (Duong et al., 2011[9]). However, although FA is considered a toxic compound for the offspring *in utero*, there is currently little evidence describing the association of these fetotoxic aspects with the maternal physiological alterations. FA is transmitted to the fetus through the placenta following maternal exposure, and its adverse effects on the fetal growth processes may be related to the degree of injuries it causes to the maternal organism (Katakura et al., 1993[19]). It was reported that FA inhalation at 20-40 ppm in Sprague-Dawley rats on gestational days (GD) 6-20 resulted in a significant reduction in weight gain in both dams and fetuses (Saillenfait et al., 1989[37]). Another study in the same animal model has indicated that FA inhalation at a range of 2-10 ppm on GD6-15 affected the maternal body weight along with a slight impact on fetal weights and ossification of the pubic and ischial bones (Martin, 1990[29]).

Earlier studies have also highlighted that FA exposure may lead to neurobehavioral and hemato-immunologic disturbances. For instance, Kilburn et al. (1985[20]) have provided evidence for some neurotoxicity signs such as lack of concentration, loss of memory, disturbed sleep, impaired balance, mood disorders, and irritability in humans after indoor exposure to FA by inhalation. Otherwise, the ingestion of FA was accompanied by severe symptoms such as lethargy, seizures, and loss of consciousness (Burkhart et al., 1990[5]). Low-level exposure to FA resulted in an immediate restless behavior with noticeable motor disabilities in rats (Boja et al., 1985[4]). In addition, mice submitted to 13 week-exposure at 40 ppm exhibited dyspnea and ataxia (Maronpot et al., 1986[28]). A recent report has pointed out the possible onset of anxiety- and depression-like behaviors along with cognitive impairments in mice exposed to low doses of inhaled FA for one week (Li et al., 2016[26]). On the other hand, discrepant findings have been reported in experimental animals regarding the hematotoxic and immunotoxic outcomes of FA. In both male and female Sprague-Dawley rats, no hematocrit or hemoglobin level changes were found after a 90-day exposure to a relatively high dose (Maronpot et al., 1986[28]). In male Wistar rats, Vargova et al. (1993[44]) indicated a significant increase in hematocrit, erythrocyte count, and hemoglobin, and a decline in mean corpuscular hemoglobin concentration following a 4-week FA gavage at 40-80 mg/kg. Orally administered or inhaled doses have also been shown to alter the antibody responses in rodents, exacerbating, therefore, the overall pathological sequelae of FA exposure (Abd-Elhakim et al., 2016[1]; Sapmaz et al., 2016[38]). However, the current research efforts pay little attention to the neurobehavioral and hemato-immune aspects of FA toxicity during pregnancy. Hence, it would be well worth investigating whether gestational FA exposure can lead to maternal changes in these aspects, which may, in turn, contribute to FA-induced fetotoxicity.

Nowadays, the search for natural medicines with potential health benefits against a variety of toxic agents is on trial. A substantial interest is particularly devoted to assessing the preventive effects of bioflavonoids that are widely available for dietary intake in humans. Hesperidin (HP) (hesperetin-7-rutinoside), one of the most biologically active compounds in the flavonoid family, is a flavanone glycoside found at high levels in citrus fruits such as lemons, limes, and oranges. It has been reported to exhibit a wide range of pharmacological effects, including anticarcinogenic, anti-inflammatory and antioxidant activities (Garg et al., 2001[15]). Recent toxicological studies have highlighted the strong ability of HP to protect various tissues against the oxidative injuries caused by toxic agents (Çetin et al., 2016[7]; Siddiqi et al., 2015[40]). This bioflavonoid was also proven to alleviate the hemato-immune toxicity induced by a chronic oral exposure to diazinon in rats (Hassouna et al., 2015[16]) and restore the acquired immune activities in irradiated mice (Lee et al., 2011[25]). Furthermore, neuroprotective and cognitive-enhancing effects of HP were demonstrated in a murine model of aluminum-induced neurotoxicity (Jangra et al., 2015[18]). However, to our knowledge, no previous studies describing the perigestational intake of HP in intoxicated rodents were performed. The aforementioned findings of HP properties prompt us to explore its effectiveness in counteracting FA-related toxicity in pregnant dams and their fetuses.

This study aims to investigate the ability of perigestational treatment with HP to prevent the pathological changes in maternal behavioral, hemato-immune and hormonal parameters as well as in fetal growth signs following FA oral exposure in rats.

## Materials and Methods

### Animals and housing

Three-month-old male and female Wistar rats, obtained from Pasteur Institute, were group-housed (4-5 per cage for each sex) in transparent polyethylene cages (58 × 39 × 19 cm) in a temperature-controlled room (23 ± 1 °C). The rats were maintained on a 12-h/12-h light cycle (lights on at 07:30 a.m.) with access to standard rodent chow and tap water *ad libitum*. The experimental protocol was carried out according to the NIH revised Guidelines for the Care and Use of Laboratory Animals (No. 80-23, 1996).

### Drugs

Hesperidin (≥ 80 % purity powder; Sigma-Aldrich Co., Saint Louis, USA) was dissolved in distilled water and freshly administered by gavage at 50 mg/kg of body weight. The formaldehyde solution (37 %; Sigma-Aldrich Co., Saint Louis, USA) was prepared in distilled water and freshly administered by gavage at 2 mg/kg of body weight. Both hesperidin and formaldehyde were provided to animals in a volume of 1 ml/kg of body weight.

### Experimental procedure

After two weeks of acclimatization to the laboratory conditions, forty-four (44) female rats weighing approximately 200 ± 10 g were randomly chosen and housed individually in transparent polyethylene cages (44 × 28 × 15 cm). They were allotted to four groups (n = 11): vehicle-treated control rats (C), hesperidin-treated rats (HP), formaldehyde-exposed rats (FA), and formaldehyde-exposed rats pretreated with hesperidin (HP-FA). Rats of each group were subjected to their first vaginal smear to determine estrus cycle phases using a standard cytological analysis (Freeman, 1994[12]). Proestrus females received vehicle (groups C and FA) or HP (groups HP and HP-FA) at 09:00 a.m. Two hours later, they were treated with either vehicle (groups C and HP) or FA (groups FA and HP-FA). Both HP and FA were administered for ten (10) consecutive days (i.e. two estrus cycles) before mating. At the end of this phase, rats were subjected to the second vaginal smears, and each proestrus female was mated overnight with one sexually experienced male, without any interruption of the pharmacological administration. The presence of sperm plugs in the vaginal smears 12 hrs later indicated the first day of conception. Both HP and FA treatments were then continued until GD19. The body weight was measured daily in all pregnant females from GD1 to GD19.

At GD1, GD10 and GD19, a set of twenty-four (24) pregnant rats (six per group) were submitted to a behavioral assessment using the elevated plus-maze and open field tasks, to determine their anxiety-like and exploratory behaviors, then excluded from the experiment. The remaining twenty (20) pregnant rats (5 rats per group) were left undisturbed (except for body weight measurement) until GD19, at which they were decapitated under mild ether anesthesia. Trunk blood was collected in ethylenediaminetetraacetic acid (EDTA)-coated tubes to evaluate the hemato-immune status. Plasma samples were properly conserved and used to appraise the hormonal profile.

### Behavioral assessment

#### Elevated plus-maze test (EPM)

The EPM apparatus consisted of two open arms (50 x 10 cm) and two closed arms (50 x 10 × 45 cm) extending from a central platform (10 x 10 cm) and elevated 50 cm above the floor (Patin et al., 2005[34]). A 3-5 mm high railing was added around the perimeter of the open arms to prevent rats from falling off the maze. The test room was lit by a 60-W electric bulb hanging directly 175 cm above the central area of the maze (Estanislau and Morato, 2005[10]). Each rat was placed individually in the center of the apparatus facing one open arm and allowed to explore the maze for 5 min. The animals' behavior was videotaped and then analyzed using SMART computer software (v3.0.04., Panlab S.L.U., Spain). Upon completion of the task, the rat was returned to its home cage, and the maze was cleaned with an alcoholic solution followed by wet and dry paper towels, before the next trial.

#### Open field test (OF)

As previously described (Sáenz et al., 2006[36]), the OF apparatus consisted of a gray square arena (70 x 70 x 40 cm) with the floor divided into 16 equal quadrants by black painted lines. The test room was dimly illuminated by a red bulb (25 W) located 130 cm from the center of the arena under the same environmental conditions as the colony room. Each rat was placed individually in the center of the OF, for a free exploration of 5 minutes. The animals' activities were videotaped and recorded using SMART computer software (v3.0.04., Panlab S.L.U., Spain). After each test, the rat was removed from the arena by the experimenter and returned to the home cage, and the apparatus was cleaned with an alcoholic solution followed by wet and dry paper towels, to avoid transfer of olfactory cues between animals.

#### Hemato-immune analysis

Blood analysis was carried out using an automated cell counter (PCE-210 model 2009, Japan). Eleven parameters were measured at the same time: white blood cells (WBC), the number and percentage of lymphocytes (LYM), the number and percentage of monocytes (MONO), the number and percentage of granulocytes (GRAN), red blood cells (RBC), hemoglobin (HGB), hematocrit (HCT) and platelets (PLT).

### Hormonal analysis

Plasma levels of cortisol, 17β-estradiol (E_2_) and progesterone (P_4_) were determined by the electrochemiluminescence immunoassay (ECLIA) method using an automated analyzer according to the manufacturer's instructions (Cobas e-411 analyzer, Roche Diagnostics, Germany).

### Fetal examination

Upon sacrifice, the abdominal cavities were opened and the uteri were dissected out. The uterine horns were checked for the number of live and resorbed fetuses. Live (non-resorbed) fetuses and their placentas were carefully taken and weighed. The following morphometric parameters were measured: the cranial perimeter, the abdominal perimeter, the crown-rump length, the elbow-paw length, the foot length and the tail length. An external examination was thereafter performed to reveal any fetal malformations.

### Statistical analyses

All results are expressed as the mean ± SEM (Standard Error of the Mean). One-way analysis of variance (ANOVA) was used for multiple comparisons, followed by the Tukey-Kramer's post-hoc test when necessary. Two-way ANOVA (treatment × time) was used for behavioral tests. A linear regression analysis was applied on the maternal body weight gain. The value of *p*<0.05 was considered as the significant difference. Data were analyzed using the Minitab software (version 17.1.0.0., Minitab Ltd., UK).

## Results

### Body weight gain

**Figure 1:**
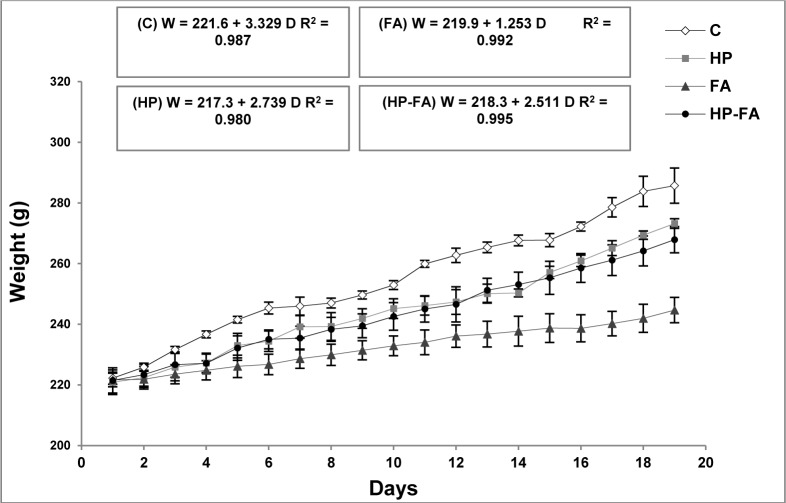
Hesperidin effect on body weight gain in FA-exposed pregnant rats. C (Control), HP (Hesperidin), FA (Formaldehyde), HP-FA (Formaldehyde-exposed rats pretreated with Hesperidin)

Figure 1(Fig. 1) depicts the regression of dams' body weight versus the gestational days. A linear relationship between weight and days was observed. In the FA-treated group, for each increase in a gestation day, the weight of the rats enhances, on the average, by 1.253 g. However, in controls, it increases by 3.329 g. Interestingly, HP supplementation in FA-treated rats remarkably ameliorates the weight gain, which increases by 2.511 g.

### Anxiety-like and exploratory behaviors in the EPM

**Figure 2:**
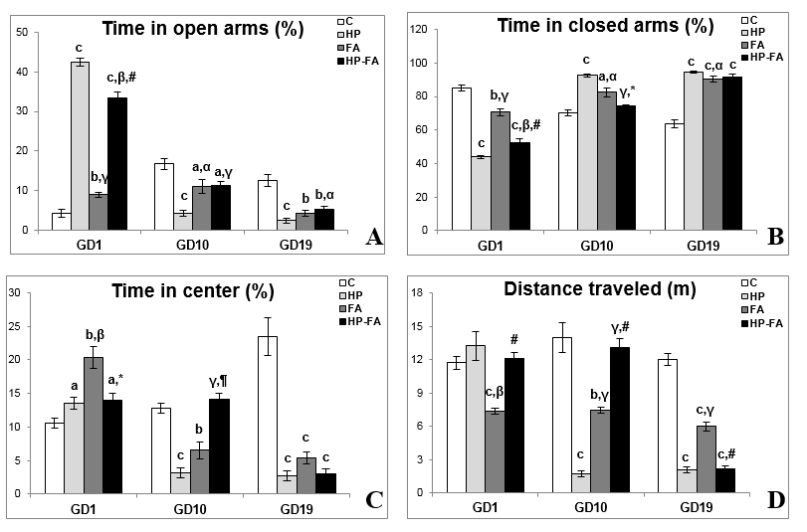
Hesperidin effect on elevated plus-maze parameters in FA-exposed pregnant rats. C (Control), HP (Hesperidin), FA (Formaldehyde), HP-FA (Formaldehyde-exposed rats pretreated with Hesperidin). a p<0.05, b p<0.01, and c p<0.001 versus C group; α p<0.05, β p<0.01, and γ p<0.001 versus HP group; * p<0.05, ¶ p<0.01, and, # p<0.001 versus FA group

Figure 2(Fig. 2) illustrates the rats' behavior in the EPM test. Two-way ANOVA revealed significant effects of treatment, time and treatment × time interaction (p<0.001) on all parameters. At GD1, HP, FA, and HP-FA groups spent shorter time in closed arms (p<0.001, p<0.01 and p<0.001, respectively), and greater time in both open arms (p<0.001, p<0.01 and p<0.001, respectively) and central area (p<0.05, p<0.01 and p<0.05, respectively) as compared to controls. Additionally, when compared to HP group, FA- and HP-FA-treated rats spent less time in the open arms (p<0.001 and p<0.01, respectively), and greater time in closed ones (p<0.001 and p<0.01, respectively). However, only FA-exposed rats spent higher time in the central zone (p<0.01). HP administration in FA-treated dams significantly enhanced the time spent in open arms (p<0.001) while decreasing the time spent in both closed arms (p<0.001) and central area (p<0.05) as compared to FA group. On the other hand, FA treatment significantly declined the distance traveled in the EPM as compared to C and HP groups (p<0.001 and p<0.01, respectively). HP supplementation in FA-exposed rats significantly enhanced the distance traveled in the apparatus (p<0.001) as compared to that in FA group. At GD10, HP and FA treatments significantly decreased the time spent in both open arms (p<0.001 and p<0.05, respectively), and central area (p<0.001 and p<0.01 respectively), and the distance traveled in the EPM (p<0.001 and p<0.01, respectively), whereas, they increased the time spent in closed arms (p<0.001 and p<0.05 respectively) as compared to controls. Furthermore, FA- and HP-FA-treated rats spent greater time in the open arms (p<0.05 and p<0.001, respectively), and shorter time in closed ones (p<0.05 and p<0.001, respectively), and traveled a longer distance in the apparatus (p<0.001) as compared to HP group. HP-FA-treated dams spent lesser time in the open arms (p<0.05) when compared to controls and longer time in the central zone (p<0.001) as compared to HP group. On the other hand, they spent a shorter time in closed arms (p<0.05) and greater time in the central area (p<0.01), and traveled a longer distance in the EPM (p<0.001) when compared to FA-exposed rats. At GD19, HP, FA and HP-FA treatments significantly declined the time spent in open arms (p<0.001, p<0.01 and p<0.01, respectively), and central zone (p<0.001) and the distance traveled in the apparatus (p<0.001), however, they enhanced the time spent in closed arms (p<0.001) as compared to controls. Interestingly, the FA-treated rats spent a shorter time in closed arms (p<0.05) and traveled a longer distance in the EPM (p<0.001) when compared to HP group. HP-FA dams spent greater time in the open arms (p<0.05) as compared to HP ones and traveled lesser distance in the apparatus (p<0.001) when compared to FA group.

### Anxiety-like and exploratory behaviors in the OF

**Figure 3:**
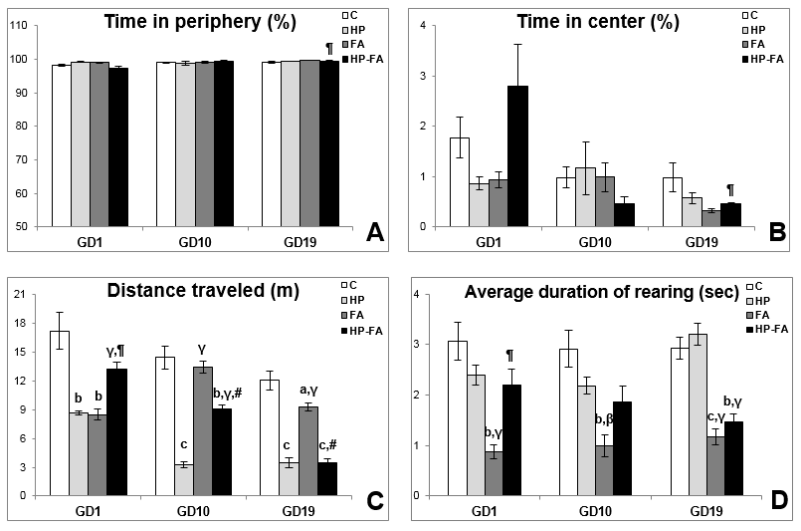
Hesperidin effect on open field parameters in FA-exposed pregnant rats. C (Control), HP (Hesperidin), FA (Formaldehyde), HP-FA (Formaldehyde-exposed rats pretreated with Hesperidin). a p<0.05, b p<0.01, and c p<0.001 versus C group; β p<0.01 and γ p<0.001 versus HP group; ¶ p<0.01 and # p<0.001 versus FA group

The rats' behavior in the open field is presented in Figure 3(Fig. 3). Two-way ANOVA registered significant effects of time (p<0.001) and treatment × time interaction (p<0.01) on total time spent in both peripheral and central areas. Furthermore, it showed significant effects of treatment (p<0.001) on the average duration of rearing, and of treatment, time, and treatment × time interaction (p<0.001) on the distance traveled in the arena. At GD1, HP and FA rats traveled lesser distance in the OF (p<0.01) as compared to controls. Moreover, FA exposure significantly decreased the average duration of rearing as compared to C, HP and HP-FA groups (p<0.01, p<0.001 and p<0.01, respectively). HP-FA treatment significantly increased the distance traveled in the apparatus when compared to both HP and FA groups (p<0.001 and p<0.01, respectively). At GD10, when compared to controls, HP and HP-FA treatments significantly declined the distance traveled in the OF (p<0.001 and p<0.01, respectively). Besides, the average duration of rearing significantly decreased in FA-treated rats (p<0.01) as compared to vehicle- and HP-treated rats. Moreover, FA and HP-FA treatments significantly enhanced the distance traveled in the arena (p<0.001) as compared to HP group. Additionally, HP-FA-treated dams traveled higher distance in the OF (p<0.001) when compared to FA group. At GD19, HP- and HP-FA-exposed rats traveled less distance in the apparatus (p<0.001) as compared to controls. FA exposure significantly decreased the distance traveled in the OF when compared to both C and HP groups (p<0.05 and p<0.001 respectively). Furthermore, HP-FA dams exhibited greater time in the center and shorter time in the peripheral area when compared to FA-treated rats (p<0.01). On the other hand, HP-FA treatment significantly declined the traveled distance in the OF (p<0.001) as compared to both C and FA groups. Interestingly, the average duration of rearing significantly declined in FA- and HP-FA-treated animals when compared to both C (p<0.001 and p<0.01, respectively) and HP (p<0.001) groups.

### Hemato-immune parameters

Table 1(Tab. 1) shows the hemato-immune parameters in the control and treated groups. Compared to controls, total leukocyte count significantly decreased in all treated rats belonging to HP, FA and HP-FA groups (p<0.01, p<0.05 and p<0.001, respectively), with the lowest level registered in HP-FA rats (p<0.001) as compared to FA ones. Significant lymphopenia was observed in HP (p<0.05), FA and HP-FA (p<0.01) dams compared to C group. The nadir was noticed in HP-FA animals (p<0.01) when compared to FA ones. The percentage of lymphocytes decreased significantly in FA (p<0.01) and HP-FA (p<0.05) groups when compared to controls, with the lowest point depicted in FA-treated dams (p<0.05) as compared to HP ones. Moreover, the mean monocyte values depleted significantly in HP (p<0.01), FA and HP-FA (p<0.001) groups when compared to controls, with the nadir noticed in HP-FA rats as compared to both HP (p<0.01) and FA (p<0.05) ones. The granulocyte levels decreased significantly in HP- and HP-FA-exposed dams (p<0.001) when compared to controls, with further decrease in HP-FA animals as compared to both HP and FA groups (p<0.01 and p<0.05, respectively). However, the percentage of granulocytes increased significantly in FA- and HP-FA-treated rats when compared to both controls (p<0.001 and p<0.01 respectively) and HP group (p<0.001), with the highest level registered in FA dams (p<0.01) as compared to HP-FA ones. HP and FA treatments significantly depleted the RBC count (p<0.001) when compared to control group. On the contrary, HP-FA treatment significantly enhanced RBC levels (p<0.001) as compared to both HP and FA dams. Interestingly, hemoglobin concentration significantly depleted in HP, FA (p<0.001) and HP-FA (p<0.05) groups when compared to controls. The nadir was noticed in FA-exposed rats (p<0.001) as compared to HP and HP-FA groups, whereas the highest point, was observed in HP-FA dams (p<0.001) when compared to FA ones. Similarly, hematocrit level significantly decreased in all treated groups (p<0.001) when compared to controls, with a remarkable rise in HP-FA-exposed dams (p<0.001) as compared to HP- and FA-treated ones. Likewise, platelets significantly declined in HP (p<0.01), FA and HP-FA (p<0.001) groups when compared to controls. The nadirs were depicted in FA- and HP-FA-treated rats (p<0.001) as compared to HP-ones, with the lowest point registered in FA group (p<0.001) when compared to HP-FA one.

### Hormonal concentrations

Table 2(Tab. 2) depicts the hormonal changes in the different groups of pregnant rats. Cortisol concentration significantly increased in FA- and HP-FA-treated dams when compared to both controls and HP-treated ones (p<0.001), with a higher level in FA group as compared to HP-FA one (p<0.001). Moreover, HP significantly enhanced the E2 concentration (p<0.01), and FA reduced its level (p<0.001) when compared to controls. Additionally, FA- and HP-FA-treated rats showed a significant decrease in the E2 level (p<0.001 and p<0.01 respectively) as compared to HP group, with the lowest point registered in FA rats (p<0.001) when compared to HP-FA ones. Furthermore, no statistically significant result was noticed in respect of progesterone concentration between all groups.

### Fetal parameters

**Figure 4:**
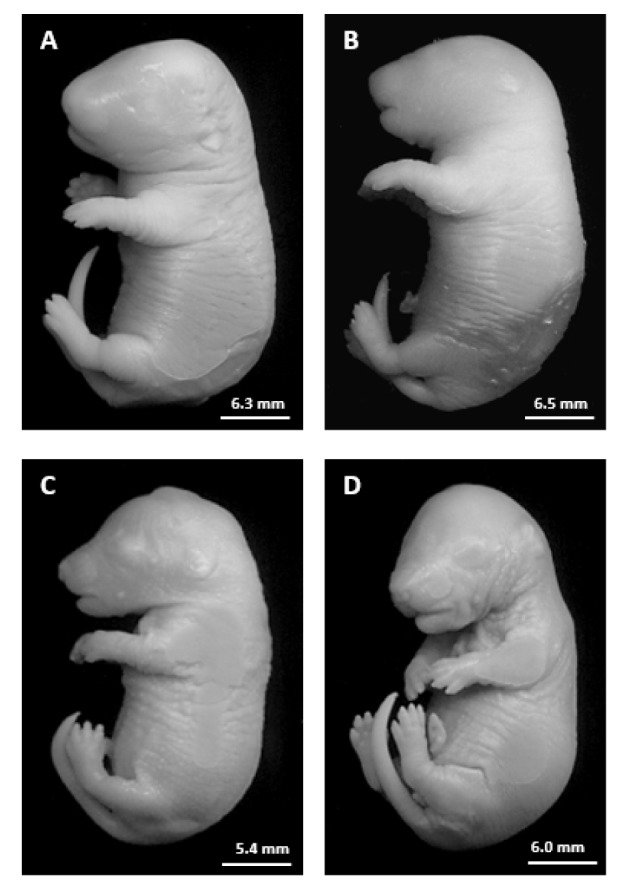
Photographs of rat fetuses from the different groups on gestational day 19. A: Fetus from C group. B: Fetus from HP group. C: Fetus from FA group. D: Fetus from HP-FA group.

Table 3(Tab. 3) demonstrates the fetal parameters in the control and treated rats. FA- and HP-FA-treated dams exhibited a lower number of the live fetuses (p<0.001) and consequently a higher number of the resorbed ones as compared to controls (p<0.001) and HP group (p<0.001 and p<0.01, respectively). HP administration in FA-exposed animals slightly enhanced the number of live fetuses (p<0.001) and reduced the number of resorption (p<0.01) when compared to FA group. Moreover, the placental weight significantly decreased in FA- and HP-FA-administered dams (p<0.05) as compared to vehicle-treated ones. The fetal body weight significantly increased in HP-treated rats (p<0.05) when compared to controls, while decreasing in FA- and HP-FA-exposed dams as compared to both controls (p<0.001 and p<0.05, respectively) and HP group (p<0.001 and p<0.01, respectively), with further decrease in FA-treated rats (p<0.01) compared to HP-FA group. Furthermore, FA exposure significantly reduced the cranial and abdominal perimeters as compared to controls (p<0.05 and p<0.001, respectively), HP and HP-FA (p<0.01) groups. The abdominal perimeter remains decreased in HP-FA fetuses (p<0.05) when compared to controls. FA treatment significantly depleted the crown-rump and elbow-paw lengths as compared to C (p<0.01 and p<0.05, respectively), HP and HP-FA (p<0.05) groups. Additionally, FA and HP-FA treatments significantly reduced the foot length (p<0.001 and p<0.05, respectively) when compared to C and HP groups, with the lowest value registered in FA fetuses (p<0.05) as compared to HP-FA ones. Similarly, FA and HP-FA treatments significantly depleted the tail length when compared to both C (p<0.01) and HP (p<0.001 and p<0.01, respectively) groups. No external malformations were found in the fetuses of the treated dams as compared to the control group (Figure 4(Fig. 4)).

## Discussion

The present study investigates the potential ability of hesperidin to counteract the pathophysiological outcomes of perigestational exposure to FA. Our results show that FA reduced the maternal weight gain. This reduction was accompanied by a significant hormonal imbalance on GD19. Cortisol levels were elevated, whereas E_2_ rates were declined in FA-exposed rats compared to control counterparts. Recently, a considerable weight loss has been registered in Sprague-Dawley male rats exposed to a relatively low dose of FA (5.27 ± 0.24 ppm) by inhalation, which might be due to a lack of appetite (Aydin et al., 2015[2]). Repetitive exposure to toxic compounds is considered as a chemical stress, which activates the hypothalamic-pituitary-adrenal (HPA) axis and leads to high secretion of cortisol from the adrenal glands (Friedman and Lawrence, 2002[13]). It has been reported that the stereotypical stress response includes suppression of appetite and food intake in about 30 % of human subjects (Stone and Brownell, 1994[43]). The concomitant decrease in E_2_ levels following gestational exposure to FA seems to be paradoxical because of lower E_2_ levels cause hyperphagia and enhance body weight gain (Butera, 2010[6]). Although the feeding behavior was not evaluated in our study, the maternal stress response to FA toxicity is likely to overwhelm the overeating effect of low E_2_ rates. In fact, under such stress circumstances, energy ensuing from overeating can be diverted to physiological activities aiming to eliminate the toxic agents and reestablish homeostasis, resulting in a body weight loss (Yau and Potenza, 2013[46]). On the other hand, we found that the treatment with HP alone significantly enhanced E_2_ levels and caused a noticeable decrease in maternal body weight as compared to controls. However, in combination with exposure to FA, the treatment was able to stabilize the body weight gain approximately at the level of HP-supplemented rats. Previous studies have stated that flavonoids can modulate estrogen rates in humans by interacting with the activity of key enzymes in estrogen biosynthesis, such as 17 beta-hydroxy steroid dehydrogenases (Jacobs and Lewis, 2002[17]), and high E_2_ rates were associated with food rejection during pregnancy in non-human primates (Czaja, 1975[8]). Our findings indicate that HP might cause anorexia in pregnant rats through the rise of E_2_ circulating levels, leading to a remarkable decrease in body weight. In HP-FA animals, the treatment with this flavanone reduced cortisol levels while enhancing E_2_ ones, alleviating, therefore, the stress response and leading apparently to a hormonal equilibrium for the sake of a better weight gain as compared to FA-exposed counterparts.

Rats submitted to daily exposure to FA exhibited anxiety-like behavior and locomotive disabilities. The anxiety signs were seen in the EPM test only as the gestational state progresses (i.e. GD10 and 19). On GD1, FA-exposed animals spent a longer time in both the open arms and the central square area, and a shorter time in the closed arms of EPM compared to their control counterparts. In the OF test, no difference between FA-exposed and control rats was found throughout the gestational period regarding the time spent in both central and peripheral areas. These two latest findings, which may erroneously be interpreted as a reduced anxiety, are actually due to a disorientation as revealed by the lower average duration of rearings (Fursenko et al., 2016[14]). On the contrary, the locomotive disabilities caused by FA were obviously found in both EPM and OF tests as indicated by the decreased distance traveled. It is generally known that anxiety disorders are related to disorientation, whereas lethargy is one of the main symptoms of sickness behavior (Barraclough, 1997[3]; Ridder et al., 2011[35]). These cognitive impairments have been linked to either high cortisol or lower E_2_ levels in different neuropsychiatric contexts (Kritz-Silverstein and Barrett-Connor, 2002[22]; Lara et al., 2013[24]; Sherwin, 1996[39]). Our results speculate that FA toxicity may cause a brain damage and lead to emotional and cognitive deficits in pregnant rats. The treatment with HP produced an anxiolytic action in the EPM particularly on GD1, and at a lesser extent on GD10 by affecting the time spent in closed arms and central square area. A significant amelioration of locomotion was also registered in HP-FA animals on GD1 in the OF test, and on GD1 and 10 for the EPM test. It is noteworthy that the anxiolytic effect of HP was found here to be masked by its sedative property obviously seen on GD10 and 19 in the EPM, and at all gestational timepoints in the OF, as demonstrated by the decreased distance traveled in both behavioral paradigms. Oral treatment with HP (50 and 100 mg/kg) for two weeks was reported to elicit anxiolytic and locomotive-enhancing effects in immobilization-stressed mice (Viswanatha et al., 2012[45]). Nevertheless, HP has repeatedly found to exhibit a strong sedative impact in mice (Loscalzo et al., 2008[27]; Martínez et al., 2009[30]). Therefore, our findings suggest that the anxiolytic efficacy of HP can be hidden by its sedative repercussion as the treatment progresses, which is correlated with the evolving FA-related sickness behavior in pregnant rats.

The hemato-immune status showed a significant decline in almost all parameters following both FA exposure and HP treatment. In FA-exposed rats, the absolute GRAN and relative MONO counts were not affected, but the relative GRAN count was increased. In HP-treated rats without FA exposure, no change in relative LYM, MONO, and GRAN counts was found. We further noticed that the effect of FA was stronger in decreasing HGB and PLT measures than HP. The administration of HP in FA-exposed animals was efficient in alleviating RBC, RGB, HCT, PLT and relative GRAN counts, but failed to restore WBC, LYM, MONO, GRAN, relative LYM and relative MONO measures. The ability of FA to alter the hematopoietic function was mentioned in several studies, though the data on this hematotoxicity are inconsistent. Exposure to this toxic agent was found to reduce WBC counts in humans (Kuo et al., 1997[23]). A more detailed study concluded that the immune profile of exposed workers shows increased B cells, but decreased total T cells (CD3) and T-suppressor cells (CD8), with no significant change in T-helper cells (CD4) (Ye et al., 2005[47]). High-dose exposure to FA in male rats was also reported to enhance RBC, MONO, and HGB measures while decreasing LYM counts (Vargova et al., 1993[44]). In our study, the immunosuppressing effect of FA could also be attributed to hypercortisolemia. Contrariwise, the lowering effect of HP on the hemato-immune status may be linked to the high E_2_ levels. Both stromal and hematopoietic cells in the bone marrow abundantly express estrogen receptors (ERs) (Smithson et al., 1995[42], 1998[41]), and the suppression of some hematopoietic lineages has been revealed in estrogen-treated adult mice (Medina and Kincade, 1994[31]; Medina et al., 2000[32]). Interestingly, HP supplementation in FA-exposed rats significantly restored the hematological part of the hemato-immune status, but failed to relieve the immunotoxicity, except for the relative GRAN count, despite the considerable attenuation of cortisol levels. These findings reflect the detrimental impact of FA on the immune system and highlight the complex HP-FA interactions to reduce toxicity in pregnant rats.

A significant reproductive failure was also noticed in FA-exposed pregnant rats. Fetal body weight and morphometric measures were considerably lower than those registered in control and HP-supplemented counterparts. The placental weight was also decreased by FA toxicity compared to control animals. Although no teratological defects were found, an extremely high resorption rate was revealed, leading to a very low number of live fetuses per litter in FA-exposed rats. It was recently reported in pregnant Balb/C mice that exposure to FA during organogenesis induces toxic changes in the placental structure and functions, causing a considerable decrease in fetal weights (Monfared, 2014[33]). Nevertheless, our findings further indicate that these reproductive deficits are probably related to FA-induced hormonal changes because HP co-administration, by regulating the hormonal status, was able to alleviate almost all developmental parameters without restoring the placental weight to control levels. To our knowledge, there are no published data about the interference of HP supplementation with toxic repercussions of FA exposure in pregnant subjects. 

Our study showed that perigestational exposure to FA was associated with various toxicological aspects in rats. Significant changes in hormonal (except for P_4_ rates) and hemato-immune profiles, along with a decrease in body weight gain and a behavioral alteration (anxiety- and lethargy-like signs, disorientation and locomotor disabilities), were found in dams. Severe developmental deficits were also registered in FA-exposed offspring at GD19, as indicated by lower fetal body weight, fetal morphometric measures, and placental weight as well as by higher resorption rates. However, no fetal external malformations were seen. Co-administration of HP was able to sustain the maternal body weight gain and regulate the hormonal status while alleviating FA-induced hematological decline. However, the immunotoxicity caused by FA was not relieved by HP treatment. The anxiolytic and motor-enhancing effects of HP were revealed at GD1 and 10 but hidden later by a strong sedative impact, which might evolve as the treatment duration progresses. In the offspring, the treatment with HP mitigated FA-induced developmental deficits without affecting the placental weight. It is noteworthy that, besides a sedative effect, HP supplementation was accompanied by higher E_2_ plasma levels in the positive control animals (group HP), with no adverse repercussions on both dams and offspring. Similar investigations are needed to further evaluate the potential beneficial role of HP in counteracting FA toxicity during pregnancy.

## Conflict of interest

The authors declare that they have no conflict of interest.

## Funding

This research received no specific grant from any funding agency in the public, commercial, or not-for-profit sectors.

## Acknowledgement

We are grateful to Mounir Merzoug and Prof. Abdelatif Boutefnouchet for technical assistance.

## Figures and Tables

**Table 1:**
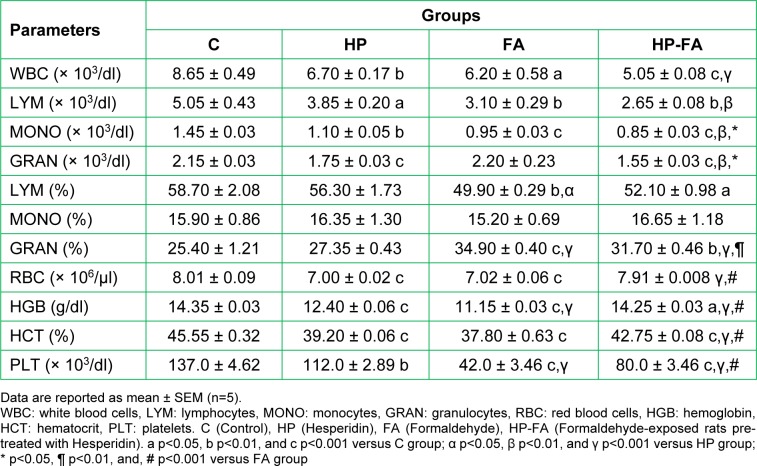
Hesperidin effect on hemato-immune parameters in FA-exposed pregnant rats

**Table 2:**
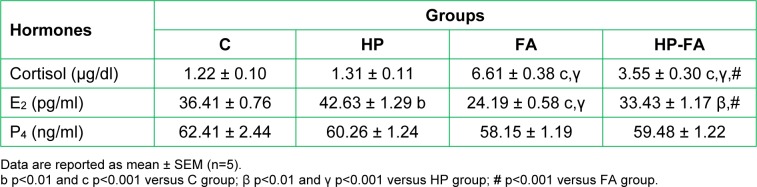
Hesperidin effect on plasma cortisol, 17β-estradiol (E_2_) and progesterone (P_4_) levels in FA-exposed pregnant rats

**Table 3:**
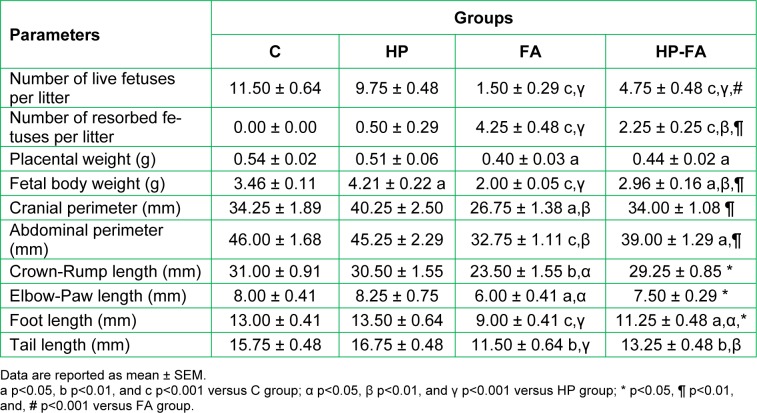
Hesperidin effect on fetal parameters in FA-exposed pregnant rats

## References

[1] Abd-Elhakim YM, Mohamed AA, Mohamed WA (2016). Hemato-immunologic impact of subchronic exposure to melamine and/or formaldehyde in mice. J Immunotoxicol.

[2] Aydin S, Ogeturk M, Kuloglu T, Kavakli A, Aydin S (2015). Effect of carnosine supplementation on apoptosis and irisin, total oxidant and antioxidants levels in the serum, liver and lung tissues in rats exposed to formaldehyde inhalation. Peptides.

[3] Barraclough J (1997). ABC of palliative care. Depression, anxiety, and confusion. BMJ.

[4] Boja JW, Nielsen JA, Foldvary E, Truitt EB (1985). Acute low-level formaldehyde behavioural and neurochemical toxicity in the rat. Prog Neuro-Psychopharmacol Biol Psychiat.

[5] Burkhart KK, Kulig KW, McMartin KE (1990). Formate levels following a formalin ingestion. Vet Hum Toxicol.

[6] Butera PC (2010). Estradiol and the control of food intake. Physiol Behav.

[7] Çetin A, Çiftçi O, Otlu A (2016). Protective effect of hesperidin on oxidative and histological liver damage following carbon tetrachloride administration in Wistar rats. Arch Med Sci.

[8] Czaja JA (1975). Food rejection by female rhesus monkeys during the menstrual cycle and early pregnancy. Physiol Behav.

[9] Duong A, Steinmaus C, McHale CM, Vaughan CP, Zhang L (2011). Reproductive and developmental toxicity of formaldehyde: a systematic review. Mutat Res.

[10] Estanislau C, Morato S (2005). Prenatal stress produces more behavioral alterations than maternal separation in the elevated plus-maze and in the elevated T-maze. Behav Brain Res.

[11] Fischer MH (1905). The toxic effects of formaldehyde and formalin. J Exp Med.

[12] Freeman ME, Knobi E, Neill JD (1994). The neuroendocrine control of the ovarian cycle of the rat. The physiology of reproduction.

[13] Friedman EM, Lawrence DA (2002). Environmental stress mediates changes in neuroimmunological interactions. Toxicol Sci.

[14] Fursenko DV, Khotskin NV, Kulikov VA, Kulikov AV (2016). Behavioral phenotyping of mice deficient in the tumor necrosis factor. Russ J Genet Appl Res.

[15] Garg A, Garg S, Zaneveld LJD, Singla AK (2001). Chemistry and pharmacology of the citrus bioflavonoid hesperidin. Phytother Res.

[16] Hassouna I, Ibrahim H, Abdel Gaffar F, El-Elaimy I, Abdel Latif H (2015). Simultaneous administration of hesperidin or garlic oil modulates diazinon-induced hemato- and immunotoxicity in rats. Immunopharmacol Immunotoxicol.

[17] Jacobs MN, Lewis DF (2002). Steroid hormone receptors and dietary ligands: a selective review. Proc Nutr Soc.

[18] Jangra A, Kasbe P, Pandey SN, Dwivedi S, Gurjar SS, Kwatra M (2015). Hesperidin and silibinin ameliorate aluminum-induced neurotoxicity: modulation of antioxidants and inflammatory cytokines level in mice hippocampus. Biol Trace Elem Res.

[19] Katakura Y, Kishi R, Okui T, Ikeda T, Miyake H (1993). Distribution of radioactivity from 14C-formaldehyde in pregnant mice and their fetuses. Br J Ind Med.

[20] Kilburn KH, Warshaw R, Boylen CT, Johnson SJ, Seidman B, Sinclair R (1985). Pulmonary and neurobehavioral effects of formaldehyde exposure. Arch Environ Health.

[21] Kim KH, Jahan SA, Lee JT (2011). Exposure to formaldehyde and its potential human health hazards. J Environ Sci Health C Environ Carcinog Ecotoxicol Rev.

[22] Kritz-Silverstein D, Barrett-Connor E (2002). Hysterectomy, oophorectomy, and cognitive function in older women. J Am Geriatr Soc.

[23] Kuo H, Jian G, Chen C, Liu C, Lai J (1997). White blood cell count as an indicator of formaldehyde exposure. Bull Environ Contam Toxicol.

[24] Lara VP, Caramelli P, Teixeira AL, Barbosa MT, Carmona KC, Carvalho MG (2013). High cortisol levels are associated with cognitive impairment no-dementia (CIND) and dementia. Clin Chim Acta.

[25] Lee YR, Jung JH, Kim HS (2011). Hesperidin partially restores impaired immune and nutritional function in irradiated mice. J Med Food.

[26] Li Y, Song Z, Ding Y, Xin Y, Wu T, Su T (2016). Effects of formaldehyde exposure on anxiety-like and depression-like behavior, cognition, central levels of glucocorticoid receptor and tyrosine hydroxylase in mice. Chemosphere.

[27] Loscalzo LM, Wasowski C, Paladini AC, Marder M (2008). Opioid receptors are involved in the sedative and antinociceptive effects of hesperidin as well as in its potentiation with benzodiazepines. Eur J Pharmacol.

[28] Maronpot RR, Miller RA, Clarke WJ, Westerberg RB, Decker JR, Moss OR (1986). Toxicity of formaldehyde vapor in B6C3F1 mice exposed for 13 weeks. Toxicology.

[29] Martin WJ (1990). A teratology study of inhaled formaldehyde in the rat. Reprod Toxicol.

[30] Martínez MC, Fernandez SP, Loscalzo LM, Wasowski C, Paladini AC, Marder M (2009). Hesperidin, a flavonoid glycoside with sedative effect, decreases brain pERK1/2 levels in mice. Pharmacol Biochem Behav.

[31] Medina KL, Kincade PW (1994). Pregnancy-related steroids are potential negative regulators of B lymphopoiesis. Proc Natl Acad Sci USA.

[32] Medina KL, Strasser A, Kincade PW (2000). Estrogen influences the differentiation, proliferation, and survival of early B-lineage precursors. Blood.

[33] Monfared AL (2014). Histomorphological and ultrastructural changes of the placenta in mice exposed to formaldehyde. Toxicol Ind Health.

[34] Patin V, Lordi B, Vincent A, Caston J (2005). Effects of prenatal stress on anxiety and social interactions in adult rats. Dev Brain Res.

[35] Ridder DA, Lang M-F, Salinin S, Röderer JP, Struss M, Maser-Gluth C (2011). TAK1 in brain endothelial cells mediates fever and lethargy. J Exp Med.

[36] Sáenz JCB, Villagra OR, Trías JF (2006). Factor analysis of forced swimming test, sucrose preference test and open field test on enriched, social and isolated reared rats. Behav Brain Res.

[37] Saillenfait AM, Bonnet P, deCeaurriz J (1989). The effects of maternally inhaled formaldehyde on embryonal and foetal development in rats. Food Chem Toxicol.

[38] Sapmaz HI, Sarsılmaz M, Gödekmerdan A, Ögetürk M, Taş U, Köse E (2016). Effects of formaldehyde inhalation on humoral immunity and protective effect of Nigella sativa oil: An experimental study. Toxicol Ind Health.

[39] Sherwin BB (1996). Hormones, mood, and cognitive functioning in post-menopausal women. Obstet Gynecol.

[40] Siddiqi A, Hasan SK, Nafees S, Rashid S, Saidullah B, Sultana S (2015). Chemopreventive efficacy of hesperidin against chemically induced nephrotoxicity and renal carcinogenesis via amelioration of oxidative stress and modulation of multiple molecular pathways. Exp Mol Pathol.

[41] Smithson G, Couse JF, Lubahn DB, Korach KS, Kincade PW (1998). The role of estrogen receptors and androgen receptors in sex steroid regulation of B lymphopoiesis. J Immunol.

[42] Smithson G, Medina K, Ponting I, Kincade PW (1995). Estrogen suppresses stromal cell-dependent lymphopoiesis in culture. J Immunol.

[43] Stone AA, Brownell KD (1994). The stress-eating paradox: multiple daily measurements in adult males and females. Psychol Health.

[44] Vargova M, Wagnerov J, Liskova A, Jakubovský J, Gajdová M, Stolcová E (1993). Subacute immunotoxicity study of formaldehyde in male rats. Drug Chem Toxicol.

[45] Viswanatha GL, Shylaja H, Sandeep Rao KS, Santhosh Kumar VR, Jagadeesh M (2012). Hesperidin ameliorates immobilization-stress-induced behavioral and biochemical alterations and mitochondrial dysfunction in mice by modulating nitrergic pathway. ISRN Pharmacol.

[46] Yau YHC, Potenza MN (2013). Stress and eating behaviors. Minerva Endocrinol.

[47] Ye X, Yan W, Xie H, Zhao M, Ying C (2005). Cytogenetic analysis of nasal mucosa cells and lymphocytes from high-level long-term formaldehyde exposed workers and low-level short-term exposed waiters. Mutat Res.

